# Improvement of Drought Tolerance by Exogenous Spermidine in Germinating Wheat (*Triticum aestivum* L.) Plants Is Accompanied with Changes in Metabolite Composition

**DOI:** 10.3390/ijms23169047

**Published:** 2022-08-12

**Authors:** Fatemeh Gholizadeh, Tibor Janda, Orsolya Kinga Gondor, Magda Pál, Gabriella Szalai, Amirali Sadeghi, Aras Turkoglu

**Affiliations:** 1 Department of Plant Physiology and Metabolomics, Agricultural Institute, Centre for Agricultural Research, 2462 Martonvásár, Hungary; 2 Department of Plant Production and Genetics, Faculty of Agriculture, University of Kurdistan, Sanandaj 66177-15175, Iran; 3 Department of Agrotechnology, Ferdowsi University of Mashhad, Mashhad 91779-48974, Iran; 4 Department of Field Crops, Faculty of Agriculture, Necmettin Erbakan University, Konya 42310, Türkiye

**Keywords:** drought stress, wheat, seed germination, exogenous spermidine, gene expression, metabolomics

## Abstract

Drought is one of the most important environmental factors reducing the yield and production of crops, including wheat. Polyamines are closely associated with plant stress tolerance. The present study investigated the mechanisms through seed germination with spermidine protecting wheat varieties from drought stress. In the first experiment, the effects of spermidine on the germination of wheat varieties, namely Rakhshan, Mihan, Sirvan and Pishgam, were investigated in three drought levels, namely 0, −2, and −4 MPa induced by polyethylene glycol 6000. Analysis of variance indicated that spermidine, drought stress and interaction between varieties and drought stress were significant for all traits, and with severity of stress, all traits significantly decreased. In the second experiment, detailed gene expression and non-targeted metabolomics analyses were carried out using the Rakhshan and Mihan varieties after germination, with or without spermidine treatment and/or drought stress. According to the biomass parameters, the Mihan variety showed relatively better growth compared to the other variety, but the Rakhshan one showed more pronounced responses at gene expression level to exogenous spermidine than the Mihan variety. Overall, these results showed that spermidine increased the drought tolerance of wheat at the germination stage, due to specific role of polyamine metabolism in the development of effective responses under drought stress.

## 1. Introduction

Wheat (*Triticum aestivum* L.) is a main crop and staple food all over the world. However, wheat is relatively sensitive to environmental changes. Drought stress is one of the most important abiotic stresses in agriculture, which poses a serious threat to crop production in arid and semi-arid regions of the world. Drought causes osmotic stress in plants, limiting nutrient uptake and reducing metabolism [1].

Cultivation of wheat in limited water conditions restricts vegetative and reproductive growth and ultimately reduces crop quality and yield [2,3,4]. Drought significantly affects plant phenology, growth, photosynthesis, water-nutrient relationships, and respiration. Water shortage can also cause oxidative stress in plants, when reactive oxygen species (ROS) are overproduced [1,5,6]. Increasing wheat grain yield under drought conditions is a major breeding strategy. In general, seed germination is the most critical stage during seedling growth. However, this process is usually inhibited or entirely prevented by drought stress [7]. Therefore, germination rate is one of the most important indicators for assessing drought tolerance. Cultivars with a high germination rate have a better chance of growing under stress conditions. Germination is a complex physiological process that is modulated by phytohormones or physiological activators such as polyamines [8]. Polyamines (PA) are a group of small polycationic compounds with low molecular weight. They mediate the basic aspects of growth, differentiation, and cell death in almost all living organisms, including plants. They also regulate the responses to abiotic stresses [9,10,11,12], such as drought or osmotic stress, salinity, heat, and chilling by directly binding to membrane phospholipids, scavenging free radicals, osmotic adjustment and maintaining a cation-anion balance [13,14]. Research has shown that the mutant *Arabidopsis* plants, which cannot produce spermine (Spm), are hypersensitive to drought stress [15,16]. Seed priming is a promising tool to improve stress tolerance in wheat. Seed treatments with exogenous PAs may improve germination, shoot, and root lengths, and root to shoot ratio [17]. It has also been shown that soaking wheat seeds in spermidine (Spd) or Spm, but not putrescine, may alleviate the inhibition of seed germination caused by drought stress [18]. Modulation of the PA biosynthetic pathway in transgenic rice promoted the Spd concentration in plants and conferred tolerance of drought stress [19]. Moreover, exogenous Spd helped to maintain antioxidant enzyme activities in Welsh onion (*Allium fistulosum*), which can moderate the radicle scavenging system and reduce oxidative stress [20].

These studies indicate the importance of Spd for stress tolerance in plants. However, their effects in different wheat varieties under drought conditions have not been intensively studied, yet. To increase our knowledge on seed germination in response to drought stress, the aim of this study was to compare the germination ability of different wheat varieties, and to investigate the effects of one of the PAs, Spd on germination indexes of them. Furthermore, to better understand the mode of action of Spd on germination processes, we also investigated the expression levels of genes encoding enzymes involved in PA biosynthesis parallel with metabolite analyses in two wheat genotypes, under drought stress conditions.

## 2. Results and Discussion

### 2.1. Germination Experiments

In the first set of experiments, four cultivars, Mihan and Pishgam, which are new high-yielding winter varieties, and the spring varieties Rakhshan and Sirvan, were used. These are resistant to late season drought stress; however, there is no information available on how these cultivars respond to drought stress at the germination stage. Therefore, our first goal was to evaluate the drought resistance at the germination stage of these winter and spring cultivars.

**Figure 1:**
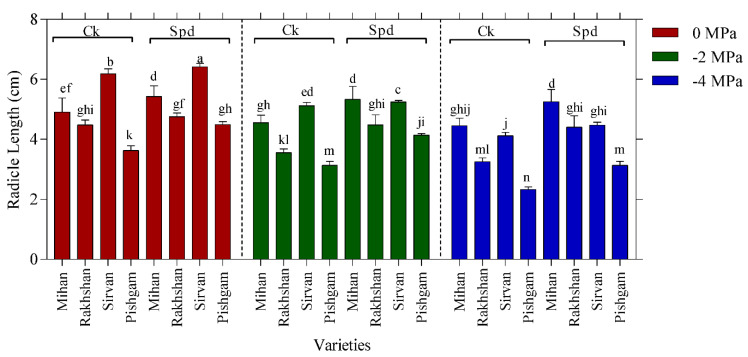
Effect of spermidine (Spd) on radicle length of four wheat varieties under drought stress with three levels of PEG 6000 osmotic potential (−2, −4 Mpa and 0 using distilled water as control). Ck = distilled water. Different letters indicate statistically significant differences across all three levels of PEG 6000 at *p* < 0.05 level using LSD’s test.

Selection of drought tolerance at early seedling stage is frequently accomplished using the simulated drought induced by chemicals like polyethylene glycol (PEG) 6000 [21]. Drought stress had a negative effect on the germination of wheat varieties according to all studied parameters. At six days after germination, the seed germination rate, germination percentage, coleoptile and radicle lengths and weights of seeds were all significantly lower for all the four varieties than the control ones. Analysis of variance results showed that there were significant differences at 1% level among wheat varieties for different levels of polyethylene glycol and Spd (Table 1). The difference between the control and Spd treatments in all varieties and drought levels shows that Spd treatment increased the stress tolerance. The results of the means comparison showed that under the control conditions, the Sirvan and Pishgam varieties could be characterized with the highest and lowest radicle lengths, respectively (Figure 1). Without PEG, the Spd treatment significantly increased the radicle lengths of the Sirvan, Pishgam and Mihan varieties, but not in the Rakhshan one. However, the effects of Spd were also significant in this variety under drought conditions. Furthermore, the use of Spd treatment in the Mihan variety under −4 MPa drought treatment increased radicle length so much that the highest radicle length was related to the Spd treated Mihan variety and the lowest was related to control treatment in the Pishgam variety (Figure 1).

**Figure 2:**
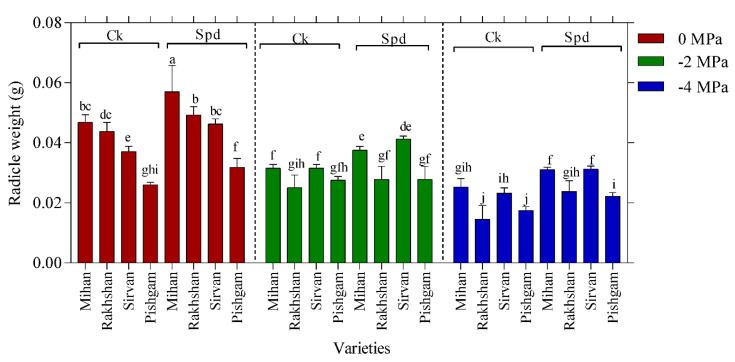
Effect of spermidine (Spd) on radicle weight of four wheat varieties under drought stress with three levels of PEG 6000 osmotic potential (−2, −4 MPa and 0 using distilled water as control). Ck = distilled water. Different letters indicate statistically significant differences across all three levels of PEG 6000 at *p* < 0.05 level using LSD’s test.

Analysis of variance showed that there were significant interactions between drought stress and varieties (*p* ≤ 0.01), varieties and Spd (*p* ≤ 0.05) on radicle weight of four wheat varieties, too (Table 1). According to these results, the highest mean radicle weight was detected in the Mihan and Sirvan varieties, whereas the lowest radicle weight was in the Rakhshan variety (Figure 2). Similar to the radicle length, Spd treatment enhanced the radicle weight at different levels of drought stress compared to the control. Radicle weight decreased significantly with increasing PEG 6000 concentration. The results showed that the highest radicle weight was in the combination of control (without PEG) and Spd treatment in the Mihan and Sirvan varieties, also the lowest radicle weight was in the combination of highest PEG 6000 concentration (−4 MPa) and without Spd treatment in the Pishgam and Rakhshan varieties (Figure 2).

**Figure 3:**
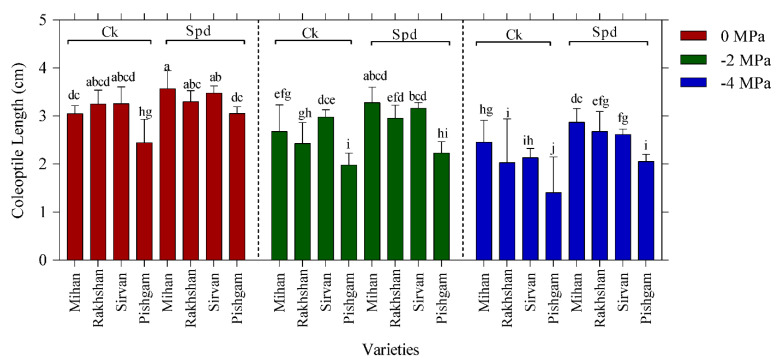
Effect of spermidine (Spd) on coleoptile length of four wheat varieties under drought stress with three levels of PEG 6000 osmotic potential (−2, −4 MPa and 0 using distilled water as control). Ck = distilled water. Different letters indicate statistically significant differences across all three levels of PEG 6000 at *p* < 0.05 level using LSD’s test.

**Figure 4:**
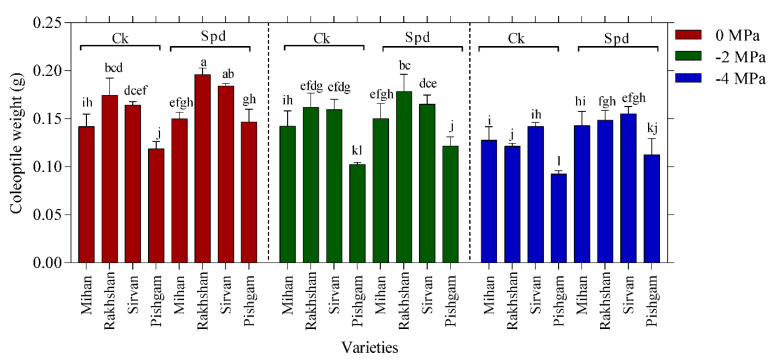
Effect of spermidine (Spd) on coleoptile weight of four wheat varieties under drought stress with three levels of PEG 6000 osmotic potential (−2, −4 MPa and 0 using distilled water as control). Ck = distilled water. Different letters indicate statistically significant differences across all three levels of PEG 6000 at *p* < 0.05 level using LSD’s test.

Analysis of variance results indicated that the interactions between variety and drought stress, Spd treatment and drought stress were significant at 1% level on coleoptile length of plants, too (Table 1). This result indicates significant differences between coleoptile length and Spd treatment with the control and other treatments under drought stress. The highest coleoptile length under drought stress was related to Spd treatment in the Mihan variety and the lowest was related to control treatment in the Pishgam variety. However, the interaction between drought stress and Spd on coleoptile length was significant, and Spd treatment had a positive effect on increasing drought resistance (Figure 3). Analysis of variance indicated that there were no significant interactions between drought stress, varieties and Spd on coleoptile weight (Table 1). With increasing PEG concentration, a clear decrease in the coleoptile weight was observed. Therefore, it can be concluded that the highest weight of coleoptile in control conditions (0 MPa) and Spd treatment was in the Sirvan variety, also the lowest weight of coleoptile was related to the highest concentration of PEG 6000 (−4 MPa) without Spd treatment in the Pishgam variety. In all varieties, Spd treatments increased the coleoptile weight (Figure 4). It has been reported that drought tolerant wheat cultivars with strong seedlings and root length are able to germinate and form seedlings in PEG 6000 solution [22,23]. Previous studies have shown that overexpression of Spd synthase increases tolerance to various abiotic stresses such as drought in transgenic *Arabidopsis thaliana*. The presence of Spd is more associated with plant stress tolerance than putrescine and Spm [24].

**Figure 5:**
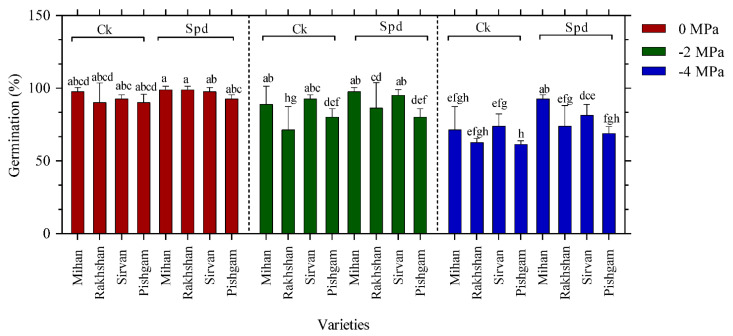
Effect of spermidine (Spd) on germination percentage of four wheat varieties under drought stress with three levels of PEG 6000 osmotic potential (−2, −4 MPa and 0 using distilled water as control). Ck = distilled water. Different letters indicate statistically significant differences across all three levels of PEG 6000 at *p* < 0.05 level using LSD’s test.

According to some reports, high Spd level was specific to meristematic cells and might be essential for the growth of global structure and root length [14,25]. Changes in germination percentage (GP) have been accepted as an important indicator of germination under drought stress [26,27]. The results of this study showed that there was a significant difference at the 5% level between drought and wheat varieties on GP (Table 1). The highest mean GP was observed in the treatment of −4 MPa in the Mihan variety with 97.50%, whereas the lowest GP was in the Pishgam variety with 68.75%. The means comparison for the highest GP in the Mihan variety was considered in different levels of drought and Spd treatments; the application of 10 mg/L Spd was 97.50%, which was higher than the control (without Spd) with 71.25% (Figure 5). These results are in accordance with other studies that increasing osmotic stress levels had negative effects on germination [26,28]. In the germination stage, fat, protein, and carbohydrate metabolism provide the necessary materials and energy for seedling growth. Furthermore, the seed germination stage and the initial seedling growth show the greatest sensitivity to drought [29]. Our results indicated Spd might be involved in the regulating of root mortality, because exogenous Spd significantly improves germination and growth of radicle and coleoptile length during germination and drought stress. It has been suggested that exogenous PAs, including Spd may increase the endogenous levels of plant hormones, which may also accelerate starch degradation in the germination seeds [27]. This may increase the concentration of soluble sugars in seeds germination processes under stress conditions. However, the effects of exogenous Spd differed in the different wheat varieties. Both the length data and the germination percentage suggest that the Mihan variety can respond to Spd more efficiently than the other varieties tested in this experiment. The significant interaction between drought stress and varieties (Table 1) also indicates that different varieties also showed different responses to drought stress. Although the interaction effect of drought and Spd was not significant in all varieties, Spd treatments often had positive effects on reducing the effect of drought stress. Results on salinity and germination showed the germinated seed number and the germination percentage had an inverse relation with salinity of substrate. Moreover, all cultivars exhibited a reduction in traits evaluated in greenhouse stage because of an increase in salinity levels [30].

**Figure 6:**
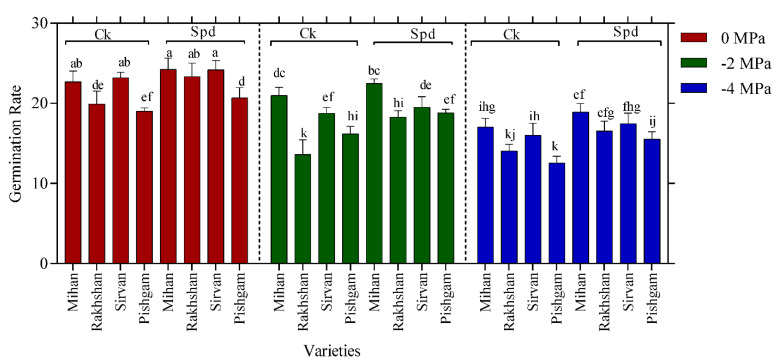
Effect of spermidine (Spd) on germination rate of four wheat varieties under drought stress with three levels of PEG 6000 osmotic potential (−2, −4 MPa and 0 using distilled water as control). Ck = distilled water. Different letters indicate statistically significant differences across all three levels of PEG 6000 at *p* < 0.05 level using LSD’s test.

The results indicated that there were significant differences among varieties and different levels of PEG, varieties and Spd treatment based on germination rate (GR). Therefore, these results for varieties showed that the highest mean GR was observed in the Mihan variety with 18.87%, whereas the lowest GR was in the Pishgam variety with 12.54% (Figure 6). Based on drought stress, Spd application in the Mihan variety gave the highest mean GR, whereas the lowest mean GR was observed in the Pishgam cultivar under −4 MPa drought stress without Spd treatment. There was a significant reduction of GR with the increased PEG concentration (Figure 6).

**Figure 7:**
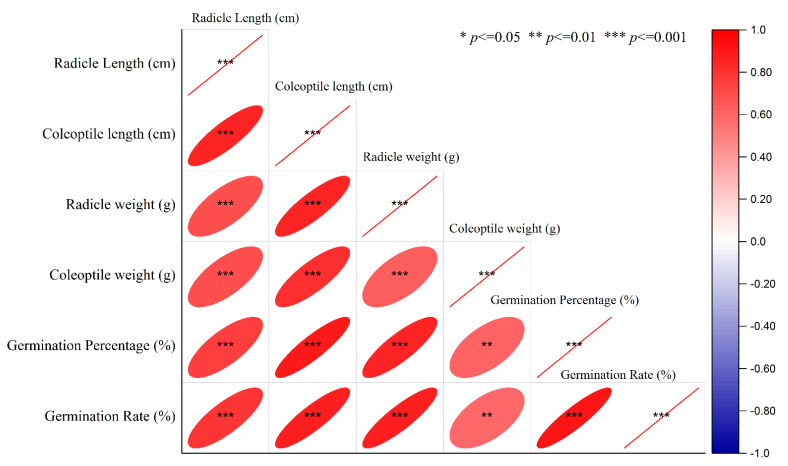
Pearson correlation between germination characteristics in wheat varieties.

**Figure 8:**
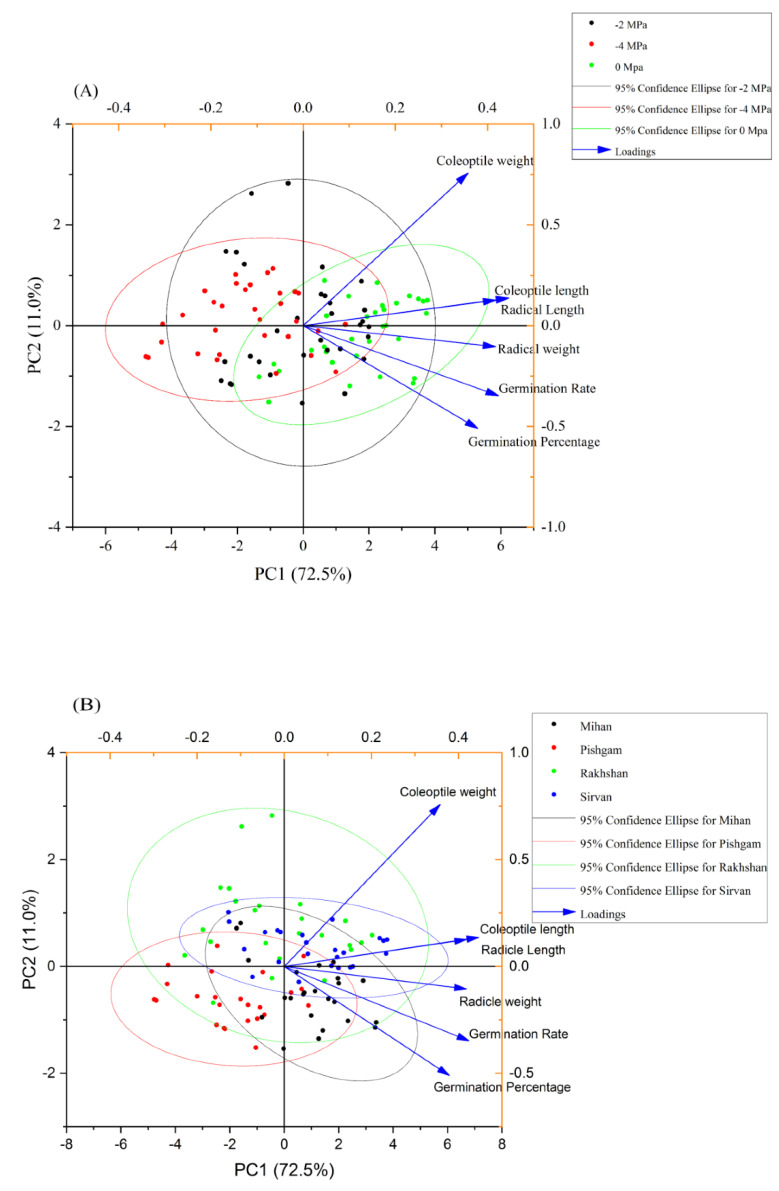
Principal component analysis for the effect of drought stress with three levels of PEG 6000 osmotic potential (−2, −4 MPa and 0 using distilled water as control) on germination and seedling growth of four wheat varieties (**A**), and for the effect of varieties on germination and seedling growth of four wheat varieties (**B**).

The correlation results showed that all growth traits were positively and significantly correlated with each other. With increasing germination, radicle and coleoptile length also had a significant positive effect on radicle and coleoptile weight of seedlings (Figure 7). Based on the results of principal component analysis, the radicle and coleoptile length of wheat seedlings were closely related (Figure 8A,B). Correlation studies between all traits showed that selection for osmotic tolerance, radicle and coleoptile length under drought stress could be helpful in predicting drought tolerance of varieties.

### 2.2. Gene Expression in Response to Drought Stress

**Figure 9:**
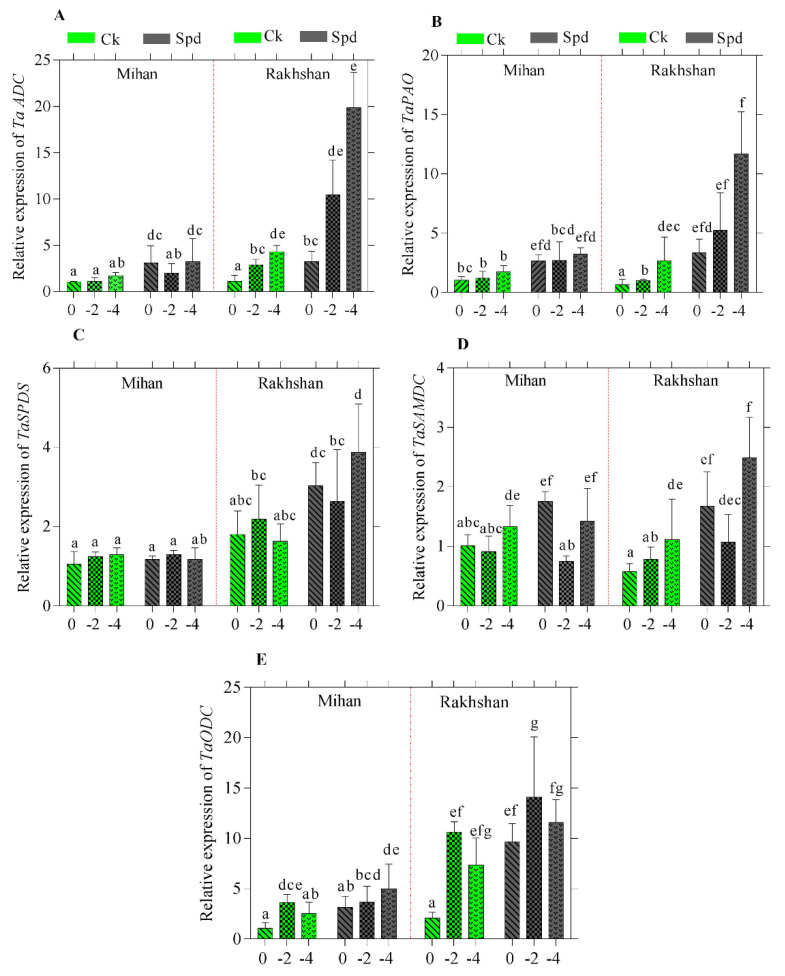
Effect of drought stress (0, −2 and −4 MPa) with Spd and Ck (control) in radicle of wheat genotypes (Mihan and Rakhshan) on relative expression of arginine decarboxylase (*TaADC*) (**A**), polyamine oxidase (*TaPAO*) (**B**), spermidine synthase (*TaSPDS*) (**C**), S-adenosylmethionine decarboxylase (*TaSAMDC*) (**D**) and ornithine decarboxylase (*TaODC*) (**E**) genes determined by qRT-PCR. All reactions for gene expression analyses were performed in triplicate using 3 biological and 3 technical repetitions. Bars show mean ± standard deviation (SD). Different letters indicate statistically significant differences at *p* < 0.05 level using LSD’s test.

In order to better understand the molecular background of the Spd action, gene expression and metabolomics analyses were carried out on two selected varieties, Mihan and Rakshan. To explore expression patterns of the genes playing role in PA synthesis and metabolism in response to drought stress, we analysed expression changes of the responsive genes under two levels of Spd (10 mg/L and distilled water as control) and three levels of PEG 6000 osmotic potential (−2, −4 MPa and distilled water as control). The expression level of genes encoding the enzymes involved in PA biosynthesis, such as ornithine decarboxylase (ODC, EC 4.1.1.17), arginine decarboxylase (ADC, EC 4.1.1.19), S-adenosylmethionine decarboxylase (SAMDC, EC 4.1.1.50), and spermidine synthase (SPDS, EC 2.5.1.16) and in PA catabolism, such as polyamine oxidase (PAO, EC 1.5.3.11) were measured. As shown in Figure 9, all of the PAO genes responsive to drought stress were found to be specific to radicles. The data shows that PEG is the most significant variable, which causes distinction in gene expression and application of PEG caused a remarkable stress in plants. In the present experiment, treatment with Spd in the Mihan and Rakhshan varieties, was in parallel with increased ADC gene expression. As shown in Figure 9A,B, the increase in ADC and PAO genes expression after Spd treatment under drought stress conditions in the Rakhshan variety was more pronounced than in the Mihan one. These results are in accordance with earlier findings in rice seedlings, where an increase in ADC activity was observed under salinity, as well as in *Arabidopsis* under low temperature and dehydration stresses [31,32]. The SAMDC expression has been shown to be induced by different abiotic stresses [33]. Additionally, the expression levels of SAMDC and Spd synthase (SPDS) genes were also significantly affected by Spd treatments in the Raksham varierty, but not in the Mihan one, where there was no significant difference between the expression of SPDS in radicle under drought stress and Spd treatment (Figure 9C,D). The activity of ODC in the radicles of the Rakhshan variety, under drought stress and Spd treatment were also significantly higher than in those of the Mihan one (Figure 9E). These results demonstrated that Spd may play an important role in the response of drought stress in wheat and suggest that the exogenously applied Spd was taken up by the wheat plants. However, it cannot be stated that there is a direct relationship between increased PA levels and stress tolerance. For example, salt tolerance was positively correlated with Spd, but negatively correlated with Spm levels [34,35]. The amount of PA is tightly regulated in the cells [10,11]. This can also be seen in the fact that although the expression levels of the genes responsible for PA synthesis were more elevated in the Raksham variety than in the Mihan variety, the PAO expression was also substantially increased, and it even showed the same pattern as that of the ADC gene. In contrast to radicle, under the present experimental conditions, PA-related gene expression levels have not been significantly induced by treatments with PEG or Spd in the coleoptile, except for ADC, which was slightly higher in the Raksham variety than in the Mihan variety after treatment with Spd (data not shown).

Overall, these results suggested the specific roles of PA synthesis and metabolism genes in the development of effective responses in wheat under drought, and that Spd increased the drought stress tolerance of wheat in the germination stage. Studies in different plant species have also shown that PAs play predominant roles in responding to environmental stresses. In addition, the application of exogenous PAs can improve resistance to diverse stresses in plants. In tomato seedlings, Spd triggers effective protection under salinity-alkalinity stress, probably by maintaining the structural integrity of chloroplasts and alleviating oxidative damage. It has been suggested that exogenous Spd may activate the antioxidant defence system and proline metabolism to protect white clover from water stress. The PAs were also reported to enhance the tolerance to heat stress [14,35,36,37,38].

**Figure 10:**
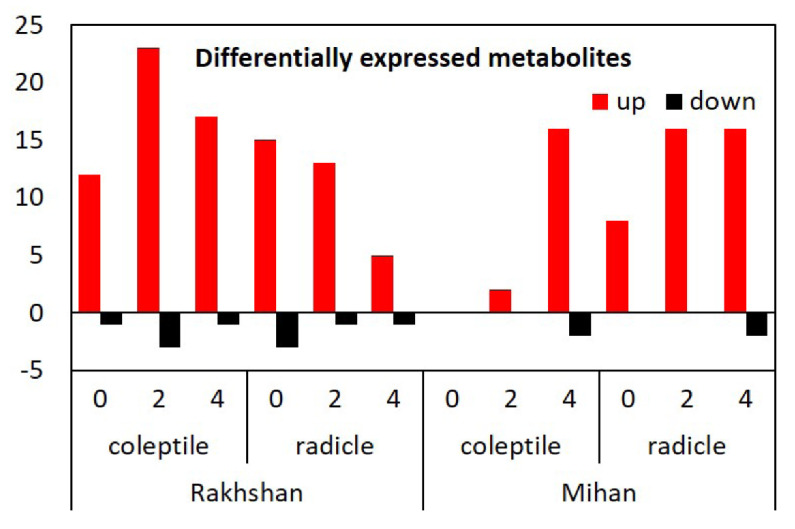
Number of the differentially expressed metabolites in two varieties (Mihan and Rakshan) under PEG 6000 treatments. 0, 2, and 4 indicates control, −2 MPa, and −4 MPa PEG treatments, respectively. Up: statistically significant increased, down: statistically significant decreased levels.

**Figure 11:**
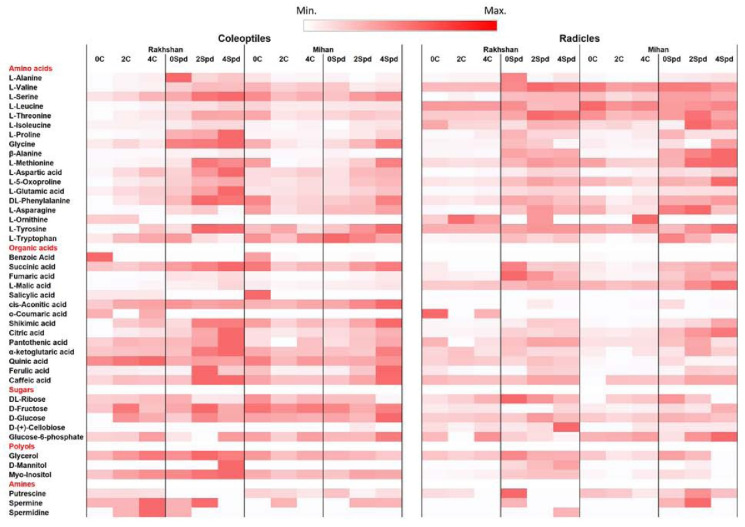
The contents of metabolites found in an untargeted analysis in the coleoptiles and radicles of two wheat varieties Rakhshan and Mihan, with or without (C) treatment with exogenous Spd. 0: control plants treated with distilled water, 2 and 4 represents PEG treatments at −2 and −4 MPa, respectively. Darker red colour indicates higher abundance of the given metabolite.

To get further insight into the background of Spd treatment, a non-targeted metabolomics analysis was carried out in the radicle and coleoptile of the Mihan and Rakshan varieties with or without exposure to PEG 6000. Forty-three metabolites were analysed, which were above the detection limit (Table S1). They were divided into five groups. Most of them, 18, belonged to amino acids, but 14 organic acids, 5 sugars, and 3-3 polyols and PAs, namely putrescine, Spd and Spm could also be detected. Most of them were upregulated by the treatment with Spd, regardless of the PEG treatments (Figure 10). However, under control, non-stressed conditions, treatment with Spd was much more pronounced in the Rakhshan than in the Mihan variety, where Spd mainly induced changes in metabolite compositions under exposure to PEG. Furthermore, the effects of exogenous Spd were much more pronounced in both varieties than the differences between the varieties or the effects of treatments with PEG (Figure 11).

Metabolites, which showed the most pronounced increases (at least 3 times) after treatment with Spd, and occurred in at least two comparisons were L-Tryptophan (in the Rakhshan control coleoptile, and radicle, in the Rakshan −2 MPa radicle, and in the Mihan −2 MPa radicle), proline (in the Rakhshan control radicle, the Rakhshan −2 MPa coleoptile), myo-inositol (in the Rakhshan control and −2 and −4 MPa radicles, and the Mihan −2 MPa radicle), alanine (in the Rakhshan −2 and −4 MPa radicles, and the Mihan −2 and −4 MPa radicles), and citric acid (in the Rakhshan 4 coleoptile and in the Mihan −2 and −4 MPa radicles). Although the used untargeted GCxGC-TOF analysis is not the most sensitive method specifically for Pas, some of them could also be detected in the wheat samples. The Spd treatment also affected the level of certain PAs. For example, a substantial increase was detected in the level of putrescine in the radicle of the Rakhshan variety under non-stressed conditions (Table S1). This is in accordance with the increased expression level of the TaODC gene, but accumulation of putrescine can also be a result of the back conversion of higher PAs due to high DAO and/or PAO activities (Figure 9B). Although treatment was carried out with Spd, substantial increase in Spd content could not be generally detected. This is not surprising, because PA levels are strictly regulated in the cells [10,11].

Shikimic acid also tended to a slight increase after treatment with Spd (Table S1). This compound serves as a starting point of various metabolomic pathways, including phenylpropanoid biosynthesis or synthesis of certain amino acids. As salicylic acid and one of its putative precursors, benzoic acid, usually tended to decrease, but several amino acids were usually increased, it suggests that Spd treatment generally at least partly down-regulated the phenylpropanoid and up-regulated the amino acid synthesis pathways. Among amino acids, the role of proline in stress acclimation has been widely studied and described. It may also serve as osmoprotectant and signalling molecule inducing other protective mechanisms under various abiotic stress conditions [39,40,41].

The role of myo-inositol, which was also abundant in Spd-treated plants, has also been studied in plants under stress conditions. Due to its special structure, myo-inositol can be bound with both lipids and phosphates, leading to special signalling messages. For example, inositol (1,4,5)-trisphosphate can stimulate intracellular Ca^2+^ release, which in turn may also trigger other signalling routes. This process is reversible because inositol polyphosphate 5-phosphatase enzyme may hydrolyse phosphates from the molecule [42]. The role of myo-inositol has been described in plants under high salinity conditions [43] or in plant–pathogen interactions [42]. Present results also suggest that it may also play role in the Spd-mediated drought tolerance in germinating wheat seedlings.

## 3. Materials and Methods

### 3.1. Plant Material and Growth Conditions

In the first set of experiments, to investigate the plant responses to Spd and drought stress, four wheat varieties (*Triticum aestivum* L.) namely, Mihan, Rakhshan, Sirvan and Pishgam, were selected, which were obtained from Agricultural Research Center of Khorasan Razavi Province, Iran. These cultivars are among the cultivars of very good quality for bakery. The Mihan and Pishgam varieties are new, high yielding winter varieties and it has been determined that they are resistant to cold stress and drought at the end of the season. The Rakhshan and Sirvan varieties are spring wheat varieties. Seeds of uniform size were sterilized by treatment with a solution of 1% sodium hypochlorite for 5 min, and then washed extensively with sterile distilled water. In this study, 25 seeds from each variety were put in each Petri dish with four repetitions. Filter paper was placed in each Petri dish with a diameter of 9 cm, and equal volume of 8 mL distilled water was added to soak the paper. Seeds soaked with Spd and PEG 6000 were placed in a germination chamber at 25 ± 1 °C for 6 days. The factors included two levels of Spd (10 mg/L and distilled water as control) and three levels of PEG 6000 osmotic potential (−2, −4 MPa and distilled water as control). The Spd concentration was based on preliminary experiments. We selected an optimum concentration, which still did not show adverse effects on the plants but was beneficial under stress conditions (Figure S1). Various osmotic potentials were prepared based on the method [44] to dissolve the required amount of PEG in distilled water at 25 °C. 6 days after the treatments, samples were harvested for measurements of the physiological traits, such as coleoptile and radicle weight, coleoptile and radicle length of seed, germination percentage (GP) and seed germination rate (GR).

All fresh radicle was immediately frozen in liquid nitrogen and stored at −80 °C for further test. After final germination, GP and GR were calculated by the following equation [45].

$$\mathrm{Germination}\;\mathrm{percentage}\;\left(\mathrm{GP}\right)=\left(\frac{\mathrm{Number}\;\mathrm{of}\;\mathrm{Total}\;\mathrm{Germinated}\;\mathrm{Seeds}}{\mathrm{Total}\;\mathrm{Number}\;\mathrm{of}\;\mathrm{Seeds}}\right)\times 100$$

$$\mathrm{Germination}\;\mathrm{Rate}\;\left(\mathrm{GR}\right)=\left(\frac{G1}{1}+\frac{G2}{2}+\frac{G3}{3}\ldots .+\frac{\mathrm{GX}}{X}\right)$$

In the second set of experiments, detailed gene expression and non-targeted metabolomics analyses were carried out using the Rakhshan and Mihan varieties in germination with or without Spd and/or drought stress.

### 3.2. Gene Expression Analysis

Total RNA was extracted from radicle samples from two wheat varieties, Mihan and Rakhshan, using TRI Reagent. The samples were treated with DNase I and cleaned with a Direct-zol™ RNA MiniPrep Kit (Zymo Research, Irvine, CA, USA) according to the manufacturer’s instructions, cDNA synthesis was carried out by using M-MLV Reverse Transcriptase (Promega Corporation, Madison, WI, USA). Gene-specific primers and housekeeping primer (Table 2), PCRBIO SyGreen Mix (PCR Biosystems, London, UK) and CFX96 Touch™ Real-Time PCR Detection System (Bio-Rad, Hercules, CA, USA) were used for quantitative real-time PCR reaction. The relative gene expression values were determined with the ΔΔCt method [46]. The Ct values were normalized by the Ct values of housekeeping gene Ta30797 encoding phosphogluconate dehydrogenase. All reactions were performed in triplicate.

### 3.3. GC-MS Analyses

Metabolomics analyses were based on methods described by [50] with some modifications. Plant samples were extracted using adonitol (60 μL of 1 mg mL^−^^1^) as internal standard. The extraction was carried out with two different concentration of methanol (60% and 90%). A 60% methanol solution was added to the samples and mixed for 30 s, used ultrasonic bath for 5 min, mixed again for 15 s and centrifuged before the supernatant was collected. This step was repeated once again using 60% and twice with 90% methanol. The supernatants were collected for analyses. A total of 100 µL of extract was dried under vacuum and formed derivative with methoxyamine hydrochloride-dissolved pyridine (20 mg mL^−1^) (Merck-Sigma group, Darmstadt, Germany) tempered at 37 °C for 90 min and with N-trimethylsilyl-N-methyl trifluoroacetamide (Merck-Sigma group, Darmstadt, Germany) tempered at 37 °C for 30 min. The samples were injected in split mode to the LECO 4D GCxGC TOFMS (LECO, St Joseph, MI, USA) at 230 °C. A 1 μL derivatized sample was injected into the 30 m column (HP-5MS ui 30 m, 0.25 mm, 0.25 μm) and the 1.5 m column (Rxi-17Sil MS phase 0.18 mm, 0.18 μm), the carrier gas (He) was used at constant flow rate (1 mL min^−^^1^). Thermal program started with 70 °C for 3 min and increased to 320 °C for 5 min in 7 °C min^−^^1^ rate. The modulation period was 3.5 s in 2D GC mode. Data evaluation and GC analyses was performed LECO ChromaTOF version 4.72 program (LECO, St Joseph, MI, USA), for identification analytical standards, Kovats retention and LECO-Fiehn metabolomics library (LECO, St Joseph, MI, USA) and NIST version 2.3 (NIST, Gaithersburg, MD, USA) databases.

### 3.4. Statistical Analyses

The experiment was conducted in a factorial arrangement based on a randomized complete block design with four replications. All data were performed by two-way analysis of variance (ANOVA) [51]. When a significant (*p* < 0.01) F ratio occurred for treatment effects, a least significant difference (LSD) was calculated. The metabolites were compared using Student’s *t*-test. Correlation analyses and principal component analyses (PCA) were assessed to determine the relationships between the traits using OriginPro 9.1 software [52].

## 4. Conclusions

The germination stage is one of the most important steps for testing drought tolerance in wheat. Drought stress delays and reduces the germination rate. Correlation coefficient and principal component analyses confirmed the negative correlation of the degree of drought stress with growth characteristics. However, significant differences for drought resistance of seed germination were observed between the different varieties. Furthermore, exogenous Spd treatment could alleviate the drought injury in wheat. It improved the germination potential of wheat varieties, not only under drought stress but also under control conditions. According to the morphological and gene expression results, the Mihan and Rakhshan varieties have the potential to survive under the increasing PEG 6000 concentrations. The Mihan variety showed relatively better growth including radicle and coleoptile length, radicle and coleoptile weight compared to the other varieties, but the Rakhshan variety showed more pronounced responses to Spd than the Mihan variety. Therefore, the Mihan and Rakhshan varieties have different strategies for overcoming drought and responded differently to the Spd treatment. The metabolomics study also showed that exogenous Spd had more pronounced effects on metabolomics composition than either the PEG-treatment or the genotype. In the long-term, the present study may also help farmers to choose drought-resistant varieties to achieve better crop productivity.

## Figures and Tables

**Table 1:** Results of variance analysis of the effect of Spd and three levels of PEG 6000 osmotic potential (−2, −4 Mpa and 0 using distilled water as control) on some morphological characteristics of wheat varieties.

Changes Sources	DF	Mean Squares					
		Radicle Length	Coleoptile Length	Radicle Weight	Coleoptile Weight	Germination Percentage	Germination Rate
Block	3	0.009 ^ns^	0.01 ^ns^	0.0003 ^ns^	0.0001 ^ns^	12.76 ^ns^	3.36 ^ns^
Drought stress (Ds)	2	13.31 **	6.14 **	0.002 **	0.002 **	2863.3 **	288.3 **
Spermidine (Spd)	1	15.01 **	4.77 **	0.0009 **	0.006 **	1544.01 **	111.9 **
Varieties (V)	3	21.39 **	3.30 **	0.0007 **	0.011 **	971.09 **	82.42 **
Spd × Ds	2	0.03 ^ns^	0.15 **	0.0001 ^ns^	0.0005 ^ns^	51.82 ^ns^	1.26 ^ns^
Ds × V	6	2.29 **	0.16 **	0.0002 **	0.001 *	276.05 **	13.78 **
V × Spd	3	0.03 ^ns^	0.09 ^ns^	0.0003 *	0.0002 ^ns^	121.78 ^ns^	6.81 **
V × Ds × Spd	6	0.20 **	0.06 ^ns^	0.0001 ^ns^	0.0001 ^ns^	193.85 *	0.267 ^ns^
Error	69	0.06	0.05	0.0009	0.0001	74.17	1.26
CV (%)		5.37	8.92	9.73	7.74	10.13	5.94

ns, * and **, non significant, significant at 5 and 1%, respectively.

**Table 2:** Primers used for analysing the expression of genes involved in biosynthesis of polyamines in wheat by quantitative real time PCR.

Gene	Forward Primer (5′-3′)	Reverse Primer (5′-3′)	Reference
*Ta30797* (PGD)	5′-GCCGTGTCCATGCCAGTG-3′	5′-TTAGCCTGAACCACCTGTGC-3′	[47]
*Ta* *ADC*	5′-TCTACCCCGTCAAGTGCAAC-3′	5′-GACGAGGCAGCTCATGGT-3′	[48]
*Ta* *ODC*	5′-CGTGCGTGGAGGTGATAGG-3′	5′-AGCTGAGGGTGCCGTAGA-3′	[48]
*TaSAMDC*	5′-ACAGCCTTCTCCACACAAGA-3′	5′-TCCAGACCAGTCATGCACA-3′	[48]
*TaSPDS*	5′-AGGTATTCAAGGGTGGCGTG-3′	5′-TGGGTTCACAGGAGTCAGGA-3′	[48]
*TaPAO*	5′-GCTCATAAATCAGCCCAATTCCA-3′	5′-TTCGCCATTTGTTGAGCTCT-3′	[49]

## Data Availability

All relevant data can be found within the manuscript and its Supplementary Materials.

## Supplementary Materials

The supporting information can be downloaded at: https://www.mdpi.com/article/10.3390/ijms23169047/s1.
