# Supplementary material for: Improvement of Drought Tolerance by Exogenous Spermidine in Germinating Wheat (Triticum aestivum L.) Plants Is Accompanied with Changes in Metabolite Composition
Source: Int J Mol Sci. 2022 Aug 12;23(16):9047. doi: 10.3390/ijms23169047 (PMC9409228; doi:10.3390/ijms23169047)
Supplement: Supplementary file 1 [file ijms-23-09047-s001.zip › ijms-1837993-supplementary.pdf]

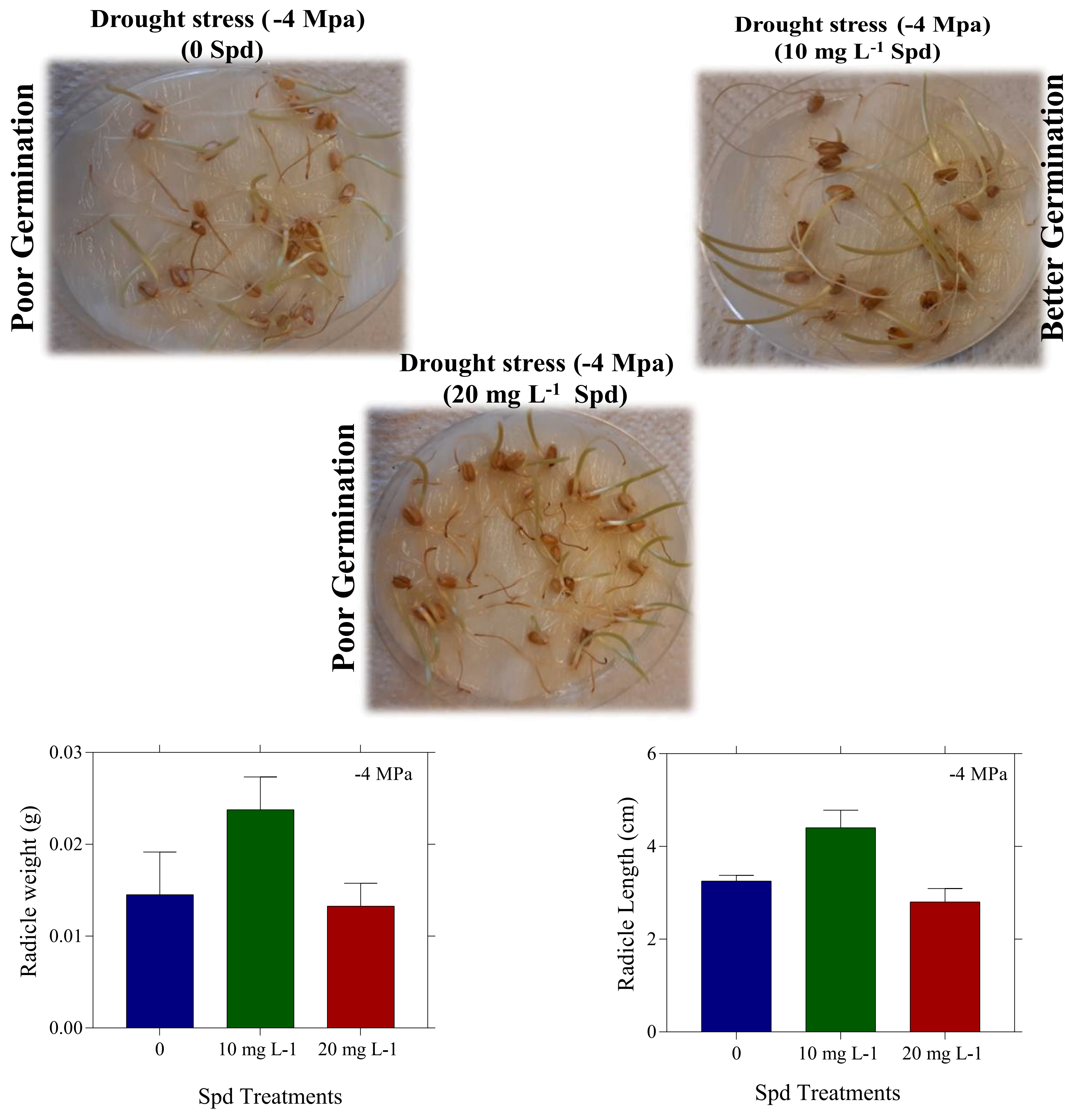
Drought stress (-4 Mpa)
(0 Spd)
Poor Germination
Drought stress (-4 Mpa)
(10 mg L-1 Spd)
Better Germination
Drought stress (-4 Mpa)
(20 mg L-1 Spd)
Poor Germination
-4 MPa
Radicle weight (g)
0.00
0.01
0.02
0.03
0
10 mg L-1
20 mg L-1
Spd Treatments
-4 MPa
Radicle Length (cm)
0
2
4
6
0
10 mg L-1
20 mg L-1
Spd Treatments
